# *Leishmania* Eukaryotic Initiation Factor (LeIF) Inhibits Parasite Growth in Murine Macrophages

**DOI:** 10.1371/journal.pone.0097319

**Published:** 2014-05-15

**Authors:** Olga Koutsoni, Mourad Barhoumi, Ikram Guizani, Eleni Dotsika

**Affiliations:** 1 Laboratory of Cellular Immunology, Department of Microbiology, Hellenic Pasteur Institute, Athens, Greece; 2 Laboratoire d’Epidémiologie Moléculaire et de Pathologie Expérimentale Appliquée aux Maladies Infectieuses/LR11IPT04, Institut Pasteur de Tunis- Université Tunis El Manar, Tunis-Belvédère, Tunisia; 3 Department of Microbiology, Medical School of Athens, National and Kapodistrian University, Athens, Greece; Indian Institute of Science, India

## Abstract

The leishmaniases constitute neglected global public health problems that require adequate control measures, prophylactic clinical vaccines and effective and non-toxic drug treatments. In this study, we explored the potential of *Leishmania infantum* eukaryotic initiation factor (LieIF), an exosomal protein, as a novel anti-infective therapeutic molecule. More specifically, we assessed the efficacy of recombinant LieIF, in combination with recombinant IFN-γ, in eliminating intracellular *L. donovani* parasites in an *in vitro* macrophage model. J774A.1 macrophages were initially treated with LieIF/IFN-γ prior to *in vitro* infection with *L. donovani* stationary phase promastigotes (pre-infection treatment), and resistance to infection was observed 72 h after infection. J774A.1 macrophages were also treated with LieIF/IFN-γ after *L. donovani* infection (post-infection treatment), and resistance to infection was also observed at both time points tested (19 h and 72 h) after infection. To elucidate the LieIF/IFN-γ-induced mechanism(s) that mediate the reduction of intracellular parasite growth, we examined the generation of potent microbicidal molecules, such as nitric oxide (NO) and reactive oxygen species (ROS), within infected macrophages. Furthermore, macrophages pre-treated with LieIF/IFN-γ showed a clear up-regulation in macrophage inflammatory protein 1α (MIP-1α) as well as tumor necrosis factor alpha (TNF-α) expression. However, significant different protein levels were not detected. In addition, macrophages pre-treated with LieIF/IFN-γ combined with anti-TNF-α monoclonal antibody produced significantly lower amounts of ROS. These data suggest that during the pre-treatment state, LieIF induces intramacrophage parasite growth inhibition through the production of TNF-α, which induces microbicidal activity by stimulating NO and ROS production. The mechanisms of NO and ROS production when macrophages are treated with LieIF after infection are probably different. Overall, these results indicate that LieIF is a good candidate for use as an anti-leishmanial molecule.

## Introduction

Protozoa belonging to the order Kinetoplastida and the genus *Leishmania* constitute an important group of more than 20 species and subspecies of parasites that are transmitted to human or animals by sandfly vectors [1]. These protozoa cause Leishmaniasis, a group of diseases characterised by a wide spectrum of clinical manifestations, ranging from self-healing skin ulcers (e.g. *Leishmania major)* to disfiguring mucosal lesions (e.g. *Leishmania braziliensis*), or fatal visceral infections (e.g. *Leishmania donovani*) [2].

*Leishmania spp.* have a digenetic life cycle, existing in two distinct forms, the flagellated promastigotes in the gut of their sand-fly vectors and the aflagellated amastigotes in the mammalian host. Within the mammalian host, *Leishmania* parasites reside in phagocytic cells, primarily macrophages (MΦs), the key cellular mediators of inflammatory responses, which are important for host immune protection against microbial infections [3]. Flagellated promastigotes are targeted to acidic vacuolar compartments in MΦs that have the characteristics of mature phagolysosomes, where they differentiate into the smaller aflagellated amastigote stage [4]. Aflagellated amastigotes replicate by binary fission, rupturing infected macrophages and spreading to uninfected cells, thereby initiating the onset of disease symptoms in the host [5].

Although a major function of MΦs is to protect the host from microbes, *Leishmania* parasites are able to survive and persist inside the MΦs, indicating that these parasites deploy sophisticated mechanisms to evade and modulate the host immune system for their own benefit. The fate of intracellular *Leishmania* parasites is determined by the activation status of MΦs. On one hand, “classically” activated MΦs by interferon-gamma (IFN-γ), are capable of killing parasites effectively [6]. The immunological pathway that induces parasite destruction involves the production of nitric oxide (NO) upon activation of inducible nitric oxide synthase (iNOS) and other leishmanicidal molecules, such as reactive oxygen species (ROS) [7]–[9]. NO and ROS are key players in the macrophage defence system against intracellular parasites. Moreover, chemokines, the critical mediators of leukocyte trafficking and cellular recruitment during inflammatory responses, have been reported to restrict the intracellular survival of *Leishmania* via the activation of NO mechanisms. More specifically, cysteine-cysteine (CC) chemokines, such as MIP-1α and MCP-1, have a potent antileishmanial role via the generation of NO [10]. It has been shown that in human macrophages infected with *L. infantum*, treatment with MIP-1α triggers NO release and increases leishmanicidal ability [11]. Additionally, MIP-1α is responsible for stimulating TNF-α secretion by macrophages, a cytokine well recognised as an activator of the anti-leishmanial activity of macrophages [12]. On the other hand, “alternatively” activated macrophages (by non-protective Th2 cytokines, IL-4 and IL-10) preferentially activate the arginase pathway to produce polyamines and enhance parasite persistence [4], [13], [14]. Consequently, parasite persistence within macrophages is determined by a balance between the ability of the immune response to sufficiently activate *Leishmania*-infected macrophages versus the ability of the parasite to resist the cytotoxic mechanisms of macrophage activation [9].

Therefore, the parasite molecules that influence early cytokine production may be key determinants of resistance or susceptibility to *Leishmania* infection. *Leishmania* eukaryotic initiation factor (LeIF), which is homologous to eukaryotic initiation factor eIF4A, is highly conserved among *Leishmania spp*. and is present in both the amastigote and promastigote forms [15]. It is abundant in the secretome [16] and in the exosomes [17] of stationary phase *L. donovani* promastigotes. LeIF was first described as an antigen that induces the production of the protective Th-1 type cytokines, IL-12 and IFN-γ, in human peripheral blood mononuclear cells (PBMC) from either leishmaniasis patients or normal individuals [15]. LeIF also modulates IL-12p70, TNF-α and IL-10 production by human monocyte-derived macrophages from healthy individuals [18], [19]. LeIF also was used as part of a trifusion recombinant protein vaccine, Leish 111f, which was shown to be protective in mice and hamster experimental models [20]–[22]. These recombinant proteins, when administered as a cocktail, were efficient for immunotherapy [23]. As LeIF induces secretion of IL-12, a cytokine necessary for the development of specific immunity towards a Th1 phenotype [24], [25], in presence of IFN-γ in macrophages or monocytes [18], [19], [26], we assessed in the present study the effect of *L. infantum* eukaryotic initiation factor (LieIF) and IFN-γ on *L. donovani* infection in J774A.1 macrophages by evaluating the intracellular parasite growth, and investigating possible mechanism(s) that induce resistance to *L. donovani* infection in these macrophages.

## Materials and Methods

### Cell and parasite culture

The immortalised macrophage cell line J774A.1, derived from adult BALB/cN mice (ATCC No: TIB-67), was purchased from the American Type Culture Collection (ATCC, Rockville, Maryland, USA). The J774A.1 cells were maintained in tissue flasks in complete medium RPMI-1640 (Biochrom AG, Berlin, Germany) with low content in phenol red supplemented with 2 mM L-glutamine, 10 mM Hepes, 24 mM NaHCO_3_, 50 µM of 2- mercaptoethanol, 100 U/ml penicillin, 100 µg/ml streptomycin and 10% v/v heat-inactivated foetal Bovine serum (FBS; Gibco, Paisley, UK)). The J774A.1 cells were sub-cultured in 25-cm^2^ cell culture flasks (CellStar, Greiner Bio-one, Germany) at 37°C with 5% CO2 in air. After incubation for 2 h, the flasks were washed three times with medium at 37°C to remove the unattached cells, and the remaining attached cells were used as the macrophage monolayer.

The *Leishmania donovani* parasite (strain MHOM/IN/1996/THAK35, zymodeme MON-2) was kindly provided by Dr. Ketty Soteriadou (Laboratory of Molecular Parasitology, Hellenic Pasteur Institute, Athens, Greece). Promastigotes were grown at 26 °C in complete RPMI-1640 medium (Biochrom AG, Berlin, Germany) in a cell culture flask (CellStar, Greiner Bio-one, Germany). Promastigote multiplication from the exponential to stationary phase was achieved at 26°C in a refrigerated incubator (Sanyo incubator mir-253 Electronic Biomedical, Japan) within 3 to 4 days (starting density of promastigotes 2×10^6^/ml and reaching the late stationary phase at 2×10^7^/ml). Late stationary phase parasites were centrifuged at 450 g (Beckman GPR Centrifuge) for 10 min and resuspended in RPMI-1640 culture medium supplemented with 10% FBS prior to their addition to the adhering macrophage monolayer.

### Cloning, protein expression and purification

The *LieIF* gene was amplified from *L. infantum* (MHOM/TN/88/Aymen) genomic DNA by PCR using 5′ oligonucleotide containing *SpeI* and *NdeI* sites and a 3′ oligonucleotide containing an *XhoI* site. The sequences of the oligonucleotides used for PCR were as follows:

LeIF_5′: 5′ GCGCG**ACTAGTCATATG** GCGCAGAATGATAAGATCG 3′

LeIF_3′: 5′ GCGCG**CTCGAG** CTCACCAAGGTAGGCAGCGAAG 3′

The underlined regions hybridized to the LieIF sequence and the regions shown in bold are the inserted restriction sites. The 5′ extensions (GCGCG) facilitated cleavage by the restriction enzymes.

The PCR product was purified from a 0.9% agarose gel and cloned into a Bluescript plasmid (Stratagene, La Jolla, CA, USA) cut with *SpeI* and *XhoI*. The sequence of the insert was confirmed by DNA sequencing. *LieIF* was then subcloned into a pET-22b expression vector (Novagen, San Diego, CA, USA) cut with *NdeI* and *XhoI*, and the protein was expressed from the Origami (DE3) *E. coli* strain (Novagen) and purified as previously reported mainly upon lysozyme treatment of the bacterial pellets and Ni affinity chromatography of the soluble bacterial extracts [27]. Protein concentration was determined using the Bio-Rad (Hercules, CA, USA) protein assay with bovine serum albumin (BSA) as a standard. Purity and concentration were verified using a 12% Coomassie-stained SDS PAGE gel. The identity of the protein LieIF was verified by western blot using rabbit anti-LieIF primary polyclonal antibodies (1/1000 dilution). The recombinant LieIF was tested for the absence of contaminating lipopolysaccharide (LPS) as previously described [18].

### Macrophage infection assay

J774A.1 macrophages were detached from culture flasks monolayer by scraping, washed and pelleted twice at 500 g (Beckman GPR Centrifuge, Germany) for 10 min, resuspended in complete RPMI-1640 culture medium and counted in Malassez chambers. Cell viability (97%) was determined by Trypan blue exclusion dye. Cell suspensions at an appropriate concentration (2×10^5^ cells/ml) were added to 24-well culture plates (Sarstedt, Numbrecht, Germany), and they were allowed to adhere for 2 h at 37°C under 5% CO_2_ atmosphere. Adherent J774A.1 cells were infected *in vitro* with the *L. donovani* stationary phase promastigotes at a ratio of 1∶15 as previously described [28]. At 4 h post-infection, non-internalised promastigotes were removed by washing 3 times with RPMI-1640 at 37°C. J774A.1 cells were treated for 15 h with recombinant LieIF (10 µg/ml) in combination with mouse recombinant IFN-γ (1 ng/ml) (BD Pharmengen, San Diego, USA) prior to or after infection with *L. donovani* promastigotes. Control cells were cultured and infected with promastigotes in a LieIF/IFN-γ-deficient medium for all of the procedures. We determined the effect of LieIF/IFN-γ treatment on *L. donovani* infection in J774A.1 macrophages at early (4 h and 19 h, respectively) and late (72 h) time points after infection. The effect of each treatment was determined using the alamarBlue (Invitrogen Life Technologies, NY, USA) colorimetric method as described previously [28]. The culture medium was removed at designated time points, and 50 µl of PBS (phosphate buffered saline) supplemented with 0.01% w/v sodium dodecyl sulphate (SDS) was added. After a 30 min incubation at 37°C in 5% CO_2_ atmosphere, J774A.1 cells were lysed, and the parasites were released into complete Schneider's Insect medium (Sigma, St. Louis, MO) for 24 h. AlamarBlue was then diluted to 10% with complete Schneider's medium and incubated for 18 h. The leishmanicidal activity was evaluated by correlation of the number of surviving *Leishmania* organisms with the absorbance of alamarBlue. The O.D. of lysed J774A.1 macrophages was used as negative control while the O.D. of lysed infected J774A.1 macrophages was used as positive control. In this method, the reduction of the culture medium and colour development of the reagent are proportional to cell proliferation by determining fluorescence at 570 nm using as a reference filter at 630 nm [29]. Additionally, in similar experimental conditions of infection, the pre-infection and post-infection treated J774A.1 macrophages were collected by using a cell scraper (Sarstedt, Inc. Newton, USA) and were washed twice with PBS. Finally, they were suspended in 200 µl PBS and cytocentrifuged (Cytospin 2, Shandon, UK) on a slide and stained with Giemsa's Azure Eosin Methylene Blue Solution (Merck, Germany). Intracellular amastigotes were counted in 200 macrophages in a microscope at 100×magnification (Olympus, BH, Japan). The infection rate (percentage of infected macrophages containing amastigotes/200 macrophages) and the parasite load (total number of intracellular parasites in 200 infected macrophages/200) were determined and compared in LeIF/IFN-γ and IFN-γ treated cells.

### NO synthesis assay

The LieIF/IFN-γ-induced NO synthesis by J774A.1 cells was measured as the accumulation of nitrites in cell culture supernatants for designated incubation periods using the Griess reaction (Sigma-Aldrich, USA) according to manufacturer's protocol [30]. Briefly, the J774A.1 cells were stimulated with LieIF/IFN-γ (10 µg/ml and 1 ng/ml, respectively), either pre- or post-infection, upon exposure to *L. donovani* promastigotes in a cell:parasite ratio 1∶15. As a positive control, J774A.1 cells were co-cultured with 1 µg/ml LPS and 1 ng/ml IFN-γ. At designated time points post-infection, 50 µl of supernatants were mixed with 100 µl of Griess reagent (1% sulphanilamide, 0.1% naphtylethylenediamine dihydrochloride, 3% phosphoric acid dissolved in distilled water) [31]. Absorbance was measured at 570 nm with a Dynatech Laboratories MRX spectrophotometer (Germany). The relative NO concentrations were calculated from a standard curve generated with known amounts of NaNO_2,_ and the data are expressed as the mean concentration units (ng/ml) ± SD.

### Generation of reactive oxygen species (ROS) in J774A.1 macrophages

#### LieIF/IFN-γ-mediated generation of ROS in J774A.1 cells

To measure the LieIF/IFN-γ-mediated generation of ROS in J774A.1 cells, we used flow cytometry with fluorescent probes (carboxy-H_2_DCFDA (Life Technologies, NY, USA) that permit the determination of intracellular fluorescence [32]. Briefly, J774A.1 cells were stimulated with LieIF/IFN-γ (10 µg/ml and 1 ng/ml), pre- or post-infection with *L. donovani* stationary phase promastigotes (as described in § 3). For each time point, the cells were probed with H_2_DCFDA (5 M) for 30 min at 37°C and 5% CO_2_. Hydrogen peroxide (H_2_O_2_; 1 mM) was added as the positive control group, and the cells were incubated for 15 min at 37°C and 5% CO_2_. The cells were analysed for intracellular ROS with FACS Calibur using CellQuest software (Becton-Dickinson, San Jose, CA). The data are expressed as the intensity of fluorescence (Geo Mean).

#### Effect of TNF-a on ROS production in J774A.1 cells

To determine the correlation of TNF-α production with generation of ROS in parasitized J774A.1 cells, we used flow cytometry with carboxy-H_2_DCFDA probe. J774A.1 cells were stimulated with LieIF/IFN-γ, as described above, with or without anti- mouse TNF-α monoclonal antibody (4 µg/ml) (clone: MP6-XT3, AbD Serotec, Oxford, UK). Cells were analyzed for intracellular ROS with FACS Calibur. Data are expressed by the following formula: Geo Mean  =  Geo Mean_(J774+LieIF+MON2+IFN-γ)_ – Geo Mean_(J774+MON2+IFN-γ)_.

### Isolation of RNA and RT - PCR

The total RNA of non-infected and infected (either pre-infection or post-infection treated) J774A.1 cells (3×10^6^) was extracted using an RNeasy Mini Kit (Qiagen, Germany) following the manufacturer's instructions [33]. A 1 µg aliquot of total RNA was then reverse-transcribed using M-MLV Reverse Transcriptase and the oligo(dT) 15 primer, according to the manufacturer's instructions (Promega, USA). The reverse transcription step was performed at 40°C for 60 min and 90°C for 5 min.

### MIP-1α and TNF-α analysis by real-time quantitative PCR

Real-time PCR was performed using an Exicycler 96 (Bioneer, Daejeon, Korea) with a SYBR Green PCR Master Mix (Kapa Biosystems, Boston, USA). The PCR mixture (20 µl) contained 5 pmoles of each primer, 3 µl of distilled water, 10 µl of commercial SYBR Green master mixture and 5 µl of cDNA. Primer pairs for MIP-1α (forward: TGG GTC CAA GAA TAC ATC ACT G, reverse: GAG GGA GAT GGG GGT TGA), TNF-α (forward: AGC CCA CGT CGT AGC AAA CCA CCA A, reverse: ACA CCC ATT CCC TTC ACA GAG CAA T) and GAPDH (forward: AGG TGG TGA AGC AGG CAT C, reverse: AGG CCC CTC CTG TTA TTA TGG) were derived from published work [34]; the primer pairs amplified 321 bp, 406 bp and 357 bp DNA fragments, respectively. GAPDH was used for normalisation. The samples were placed in low-profile 0.2 ml 8-tube strips (Bio-Rad, Hercules, USA) that were sealed with flat optical 8-cap strips (Bio-Rad, Hercules, USA). The thermal cycling conditions were as follows: an initial activation step for 10 min at 95°C followed by 45 cycles of denaturation for 30 sec at 95°C, annealing for 30 sec at 60°C and extension for 45 sec at 72°C. All PCR experiments were followed by a melting curve analysis to verify amplicon specificity. All expression ratios were computed via the *ΔΔCt* method. More specifically, cytokine cycle threshold (C_t_) values were normalised to GAPDH expression, as determined by ΔC_t_  =  C_t__(cytokine)_ – C_t__(GAPDH)_. Fold change in gene expression was determined by 2^−ΔΔCt^, where ΔΔC_t_  =  ΔC_t__(experimental)_ – ΔC_t__(control)_ [35]. All real-time PCR experiments were performed as 3 replicates from one experiment.

### MIP-1α and TNF-α detection by ELISA

MIP-1α and TNF-α were detected at the late time points either at pre-infected or post-infected J774A.1 macrophages. Culture supernatants were collected at the end of each incubation period and were stored at -80°C until analyzed. MIP-1α and TNF-α were determined by sandwich ELISA kits (900-K125, 900-K54; PeproTech, Rocky Hill, NJ) according to manufacturer's instructions. The concentrations of cytokines were calculated by using standard curves. Detection threshold for MIP-1α and TNF-α was 8 pg/ml and 16 pg/ml, respectively.

### Statistical analysis

The significance of the results was calculated using a nonparametric statistical test: the two-sided Mann-Whitney for comparison between two groups. The data shown are representative of at least three independent experiments and are presented as the mean ± SD. The level of statistical significance was set at p≤0.05.

## Results

### Effect of LieIF/IFN-γ pre-infection treatment on *L. donovani* growth in J774A.1 macrophages

**Figure 1:**
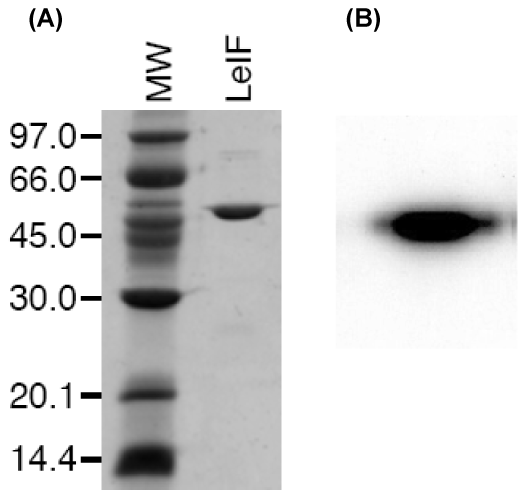
Expression and purification of the recombinant LieIF protein. (A) An aliquot of the purified LeIF was resolved by SDS polyacrylamide gel and stained with Coomassie brilliant blue. The positions of the Bio-Rad prestained marker (in kDa) are indicated at the left. (B) The identity of the protein LeIF was verified by using rabbit anti-LieIF primary polyclonal antibodies (1/1000 dilution).

**Figure 2:**
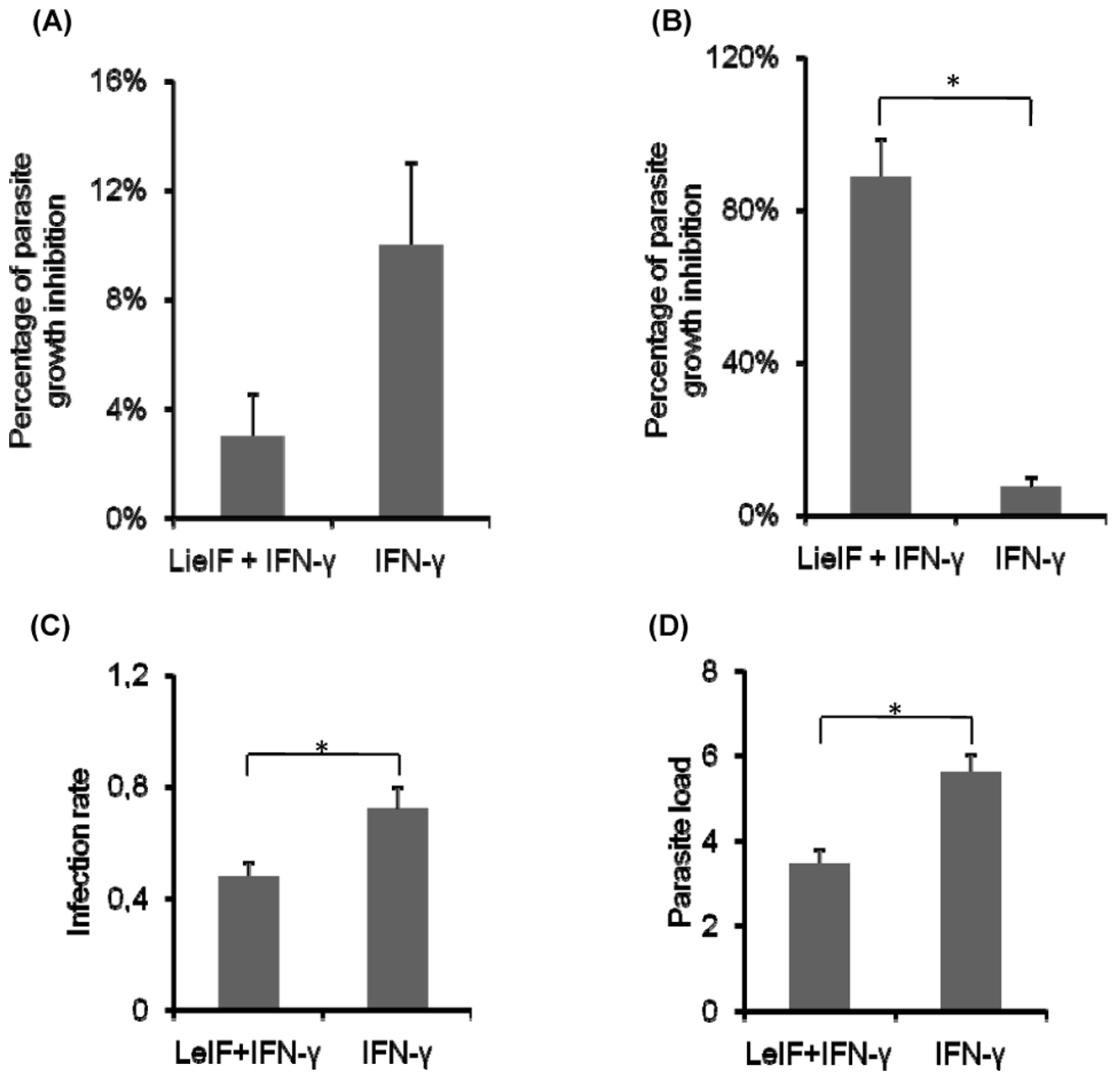
Effect of pre-infection treatment of J774A.1 macrophages with LieIF/IFN-γ on intracellular *L. donovani* growth inhibition. (A) at early (4 h) and (B) late (72 h) time points after infection. J774A.1 cells were stimulated with recombinant LieIF (10 µg/ml) and recombinant ΙFN-γ (1 ng/ml). *L. donovani* growth inhibition at the late time point (72 h) is associated with reduction of the infection rate (C) and the parasite load (D). The parasite growth inhibition was determined by the colorimetric method alamarBlue and OD was measured at 570/630 nm. Infection rate and parasite load were determined by microscopic observation upon Giemsa's staining of slides to count the mean number of infected macrophages considering 200 macrophages and the mean number of intracellular amastigotes in 200 infected macrophages, respectively. Data are presented as the mean ± S.D of at least three independent experiments. Asterisks indicate statistically significant differences (p≤0.05).

Since previous reports showed that LeIF in IFN-γ activated macrophages and monocytes induces secretion of IL-12 [18], [19], [26] a cytokine necessary for the development of specific immunity towards a Th1 phenotype [24], [25], we investigated the effect of LieIF on the susceptibility of MΦs to *L. donovani* infection. To this end, the LieIF gene was sub-cloned into pET22b plasmid containing a carboxyl terminal His 6 tag to permit the recombinant protein purification on nickel affinity column upon expression in *Escherichia coli* (Fig. 1A). The identity of the protein was verified by using rabbit anti-LieIF polyclonal antibodies (Fig. 1B). Subsequently, J774A.1 macrophages were pre-treated with LieIF/IFN-γ and were then infected with *L. donovani* parasites. The effect of these proteins was determined using the alamarBlue colorimetric method, where the number of intracellular parasites is correlated with the absorbance of the reagent. At 4 h time point after infection, the percentage of intracellular parasite growth inhibition in LieIF/IFN-γ-treated cells was low (3%), similar to the percentage obtained by IFN-γ alone (Fig. 2A). At the late time point (72 h) after infection, the LieIF/IFN-γ-treated cells showed a significantly higher inhibition of parasite growth in comparison to cells treated solely with IFN-γ (89% vs 7%) (Fig. 2B). Additionally, as shown in Fig. 2C and Fig. 2D, LieIF/IFN-γ-pre-treated macrophages exhibited significantly (p<0.05) lower *L. donovani* infection rates and parasite load than the IFN-γ-treated cells, 48% *vs* 72% and 3.48 *vs* 5.62, respectively whereas J774A.1 cells treated solely with LieIF did not exhibit parasite growth inhibition (data not shown). These results suggest that at 72 h post infection, LieIF/IFN-γ induced resistance of co-cultured J774A.1 cells to *L. donovani* infection.

### Effect of LieIF/IFN-γ post-infection treatment on *L. donovani* growth in J774A.1 macrophages

**Figure 3:**
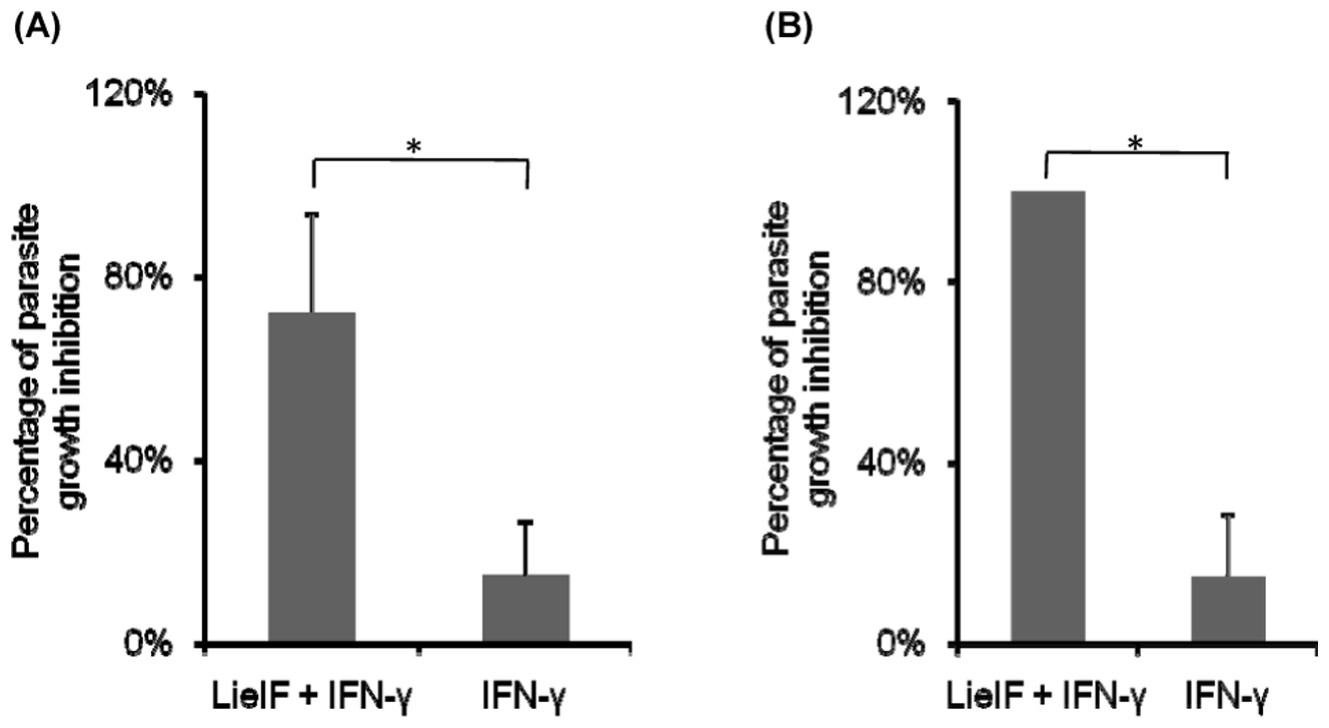
Effect of post-infection treatment of J774A.1 macrophages with LieIF/IFN-γ in intracellular *L. donovani* growth inhibition. (A) at early (19 h) and (B) late (72 h) time points after infection. J774A.1 cells were stimulated with 10 µg/ml recombinant LieIF and 1 ng/ml recombinant ΙFN-γ. *L. donovani* growth inhibition was determined by the colorimetric method alamarBlue and OD was measured at 570/630 nm. Data are presented as the mean ± S.D of at least three independent experiments. Asterisks indicate statistically significant inhibition of parasite growth (p≤0.05).

The observation that LieIF/IFN-γ-pre-treated J774A.1 macrophages showed resistance to *L. donovani* infection prompted us to examine the efficacy of treatment with LieIF/IFN-γ post- *L. donovani* infection on intracellular parasite growth. Thus, treatment of J774A.1 cells with LieIF/IFN-γ post-infection showed a statistically significant inhibition of intracellular parasite growth (p≤0.05) (Fig. 3). The inhibition of parasite growth was apparent at early (19 h) (Fig. 3A) and at late (72 h) time points after infection (Fig. 3B), with 72% and 100% parasite growth inhibition, respectively. These results suggest that J774A.1 cells post-treated with LieIF/IFN-γ also gained resistance to *L. donovani* infection, similar to their effect when administered prior to the infection.

### LieIF/IFN-γ-induced NO generation in infected J774A.1 macrophages

**Figure 4:**
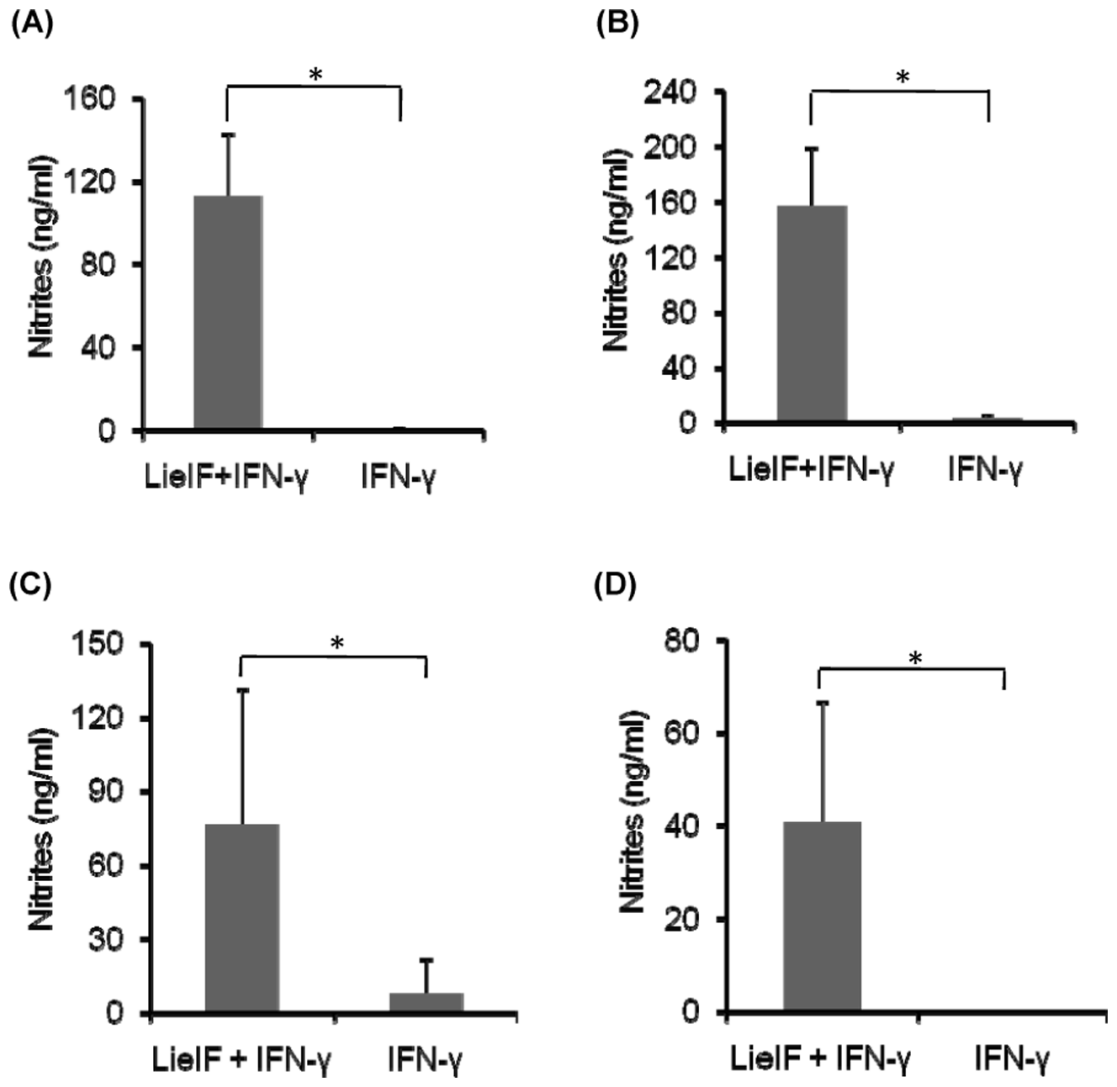
Nitric oxide production (ng/ml) by J774A.1 macrophages upon treatment with LieIF/IFN-γ at late (A, B) and early (C, D) time points after *L. donovani* infection. J774A.1 cells were treated pre-infection (A, C) and post-infection (B, D) with recombinant LieIF (10 µg/ml) and recombinant IFN-γ (1 ng/ml). Nitrite accumulation in the medium was measured by addition of Griess reagent, absorbance reading of the reaction product at 570 nm. All data are presented as the mean ± S.D of at least three independent experiments. Asterisks indicate statistically significant production of NO (p≤0.05).

The ability of J774A.1 macrophages to reduce intracellular parasite growth after treatment with LieIF/IFN-γ before or after the infection indicates that effective anti-microbial mechanisms are activated and leishmanicidal molecules are produced due to this dual treatment. Even though *Leishmania* has evolved sophisticated mechanisms leading to the repression and inhibition of NO production [36], macrophage killing of the *Leishmania* parasite *in vivo* is mediated by NO, a key mediator of nonspecific immunity. We found that infected J774A.1 cells, pre- or post-infection treated with LieIF/IFN-γ, had a marked increase in generation of NO synthesis as measured by nitrite accumulation in the culture medium determined by the Griess reaction (Fig. 4). For comparison, NO production of cells stimulated with LPS plus IFN-γ and non-stimulated cells served as positive and negative controls respectively. All presented values have been calculated after subtraction of the NO production values of the control group of infected macrophages as *L. donovani* alone is able to induce negligible amounts of NO in culture supernatants. The highest values of NO levels (113 ng/ml and 157 ng/ml) were observed, at the late time point (72 h) after infection in culture supernatants of J774A.1 cells when pre- or post-infection treated with LieIF/IFN-γ (Fig. 4A, 4B), respectively. The lowest values of NO production were detected at early time points (4 h and 19 h) after infection of J774A.1 cells that were pre- or post-infection treated with LieIF/IFN-γ, 76 ng/ml and 41 ng/ml, respectively (Fig. 4C and Fig. 4D). Collectively, LieIF together with IFN-γ induced the production of NO in infected J774A.1 cells.

### LieIF/IFN-γ triggered ROS generation in *L. donovani* infected J774A.1 macrophages

**Figure 5:**
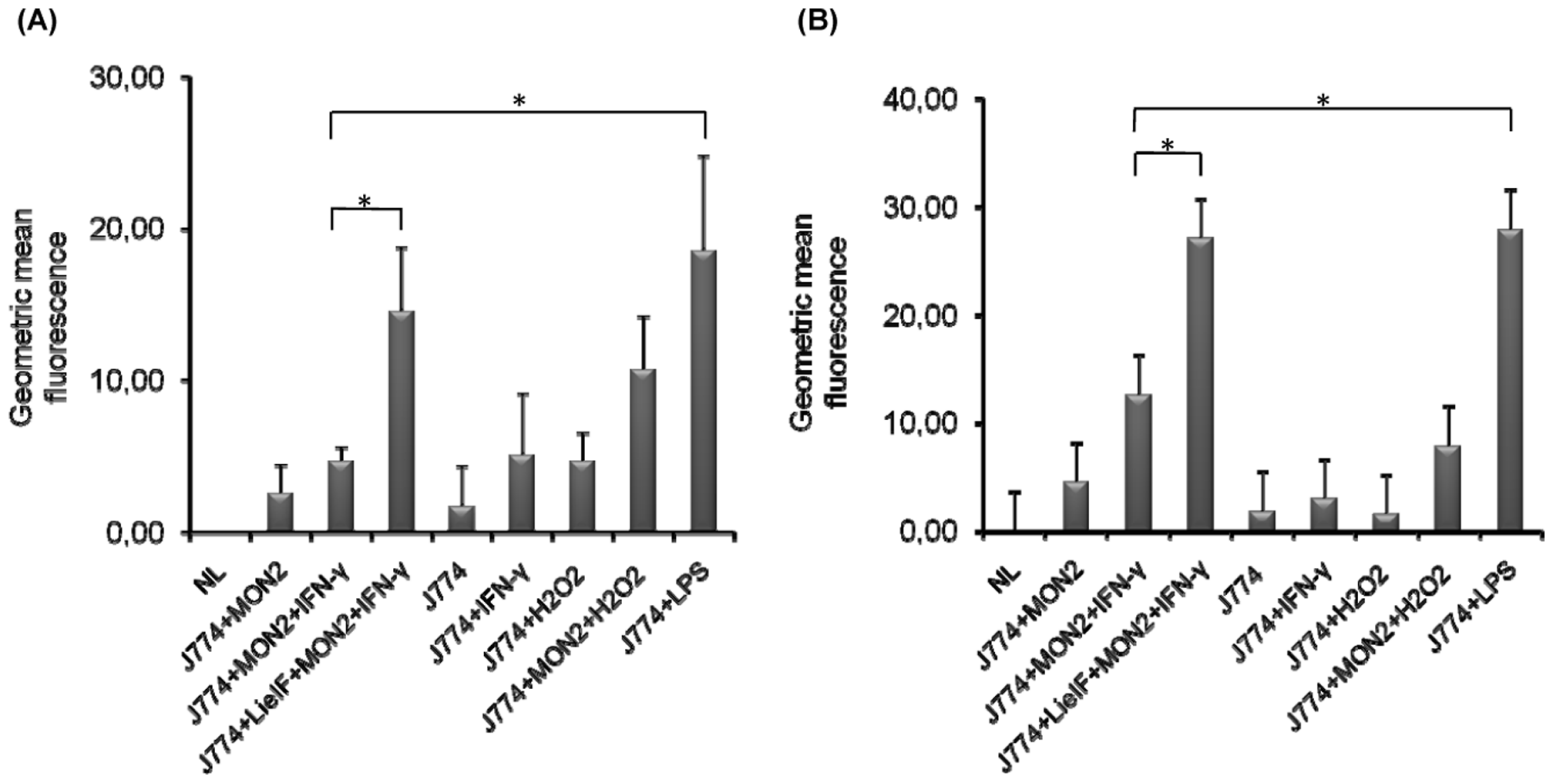
ROS generation by J774A.1 macrophages pre- and post- infection treated (A and B, respectively) with recombinant LieIF (10 µg/ml) and recombinant IFN-γ (1 ng/ml). ROS generation was determined at the late time point (72 h) after *L. donovani* infection. Cells were labelled with 5 µM H_2_DCFDA fluorescent probe and fluorescence of cells reacting with the probe was estimated by FACS analysis. All data are presented as the mean ± S.D of at least three independent experiments. Asterisks indicate statistically significant production of ROS (p≤0.05) compared to the control group of infected J774A.1 cells stimulated with IFN-γ (J774 + MON2 + IFN-γ). NL: for medium only; MON2: for the MHOM/IN/1996/THAK35 *L. donovani* strain used.

Ingestion of microbial invaders by MΦs induces the generation of reactive oxygen intermediates, which constitute an essential part of the defence mechanism adopted by the host. Because *Leishmania* parasites enter MΦs, they protect themselves against host oxidative burst through the expression of antioxidant enzymes and by the inhibition of ROS and NO production in the MΦs [37]. To investigate whether LieIF/IFN-γ caused ROS generation in J774A.1 cells, the fluorescent probe H_2_DCFDA was used. For comparison, ROS generation in LPS- and in H_2_O_2_- stimulated cells served as positive controls. The detectable inherent basal level of ROS generation in J774A.1 cells was low (Geo Mean = 1.80); thus, all the results are presented with the subtraction of the background fluorescence of non-labelled (NL) cells and are presented in Fig. 5. LieIF/IFN-γ triggered ROS generation in infected J774A.1 cells at the late time point after infection, either when added to cells pre- or post–infection, Geo Mean = 14.58 and 27.12, respectively (Fig. 5A and Fig. 5B). The ROS generation caused by addition of LieIF/IFN-γ was 3-fold higher and 2-fold higher, pre- and post-infection, respectively, than the corresponding generation caused by IFN-γ alone (Fig. 5A, 5B). Additionally, it is noteworthy that pre- and post-infection treatment with LieIF/IFN-γ produced similar amounts of ROS as those observed in the positive control (LPS-treated macrophages), 18.58 and 27.92, respectively. Interestingly, no ROS generation was evident at the early time (4 h and 19 h) points of infection (data not shown).

### LieIF/IFN-γ induced MIP-1α and TNF-α production in *L. donovani* infected J774A.1 macrophages

**Figure 6:**
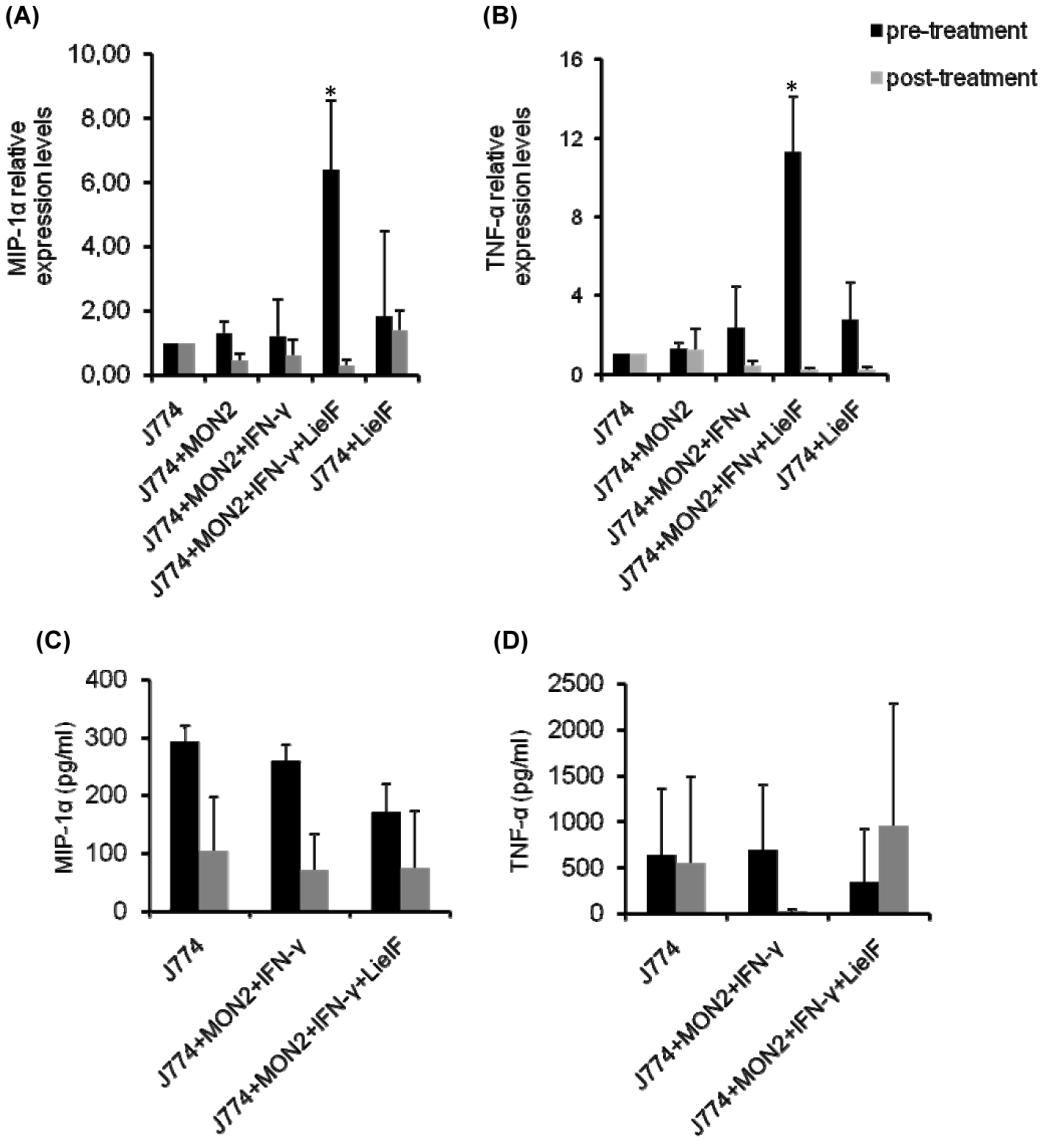
Relative expression of transcripts encoding MIP-1α (A) and TNF-α (B) in J774A.1 cells pre- (black bars) and post- infection treated (grey bars) with recombinant LieIF/IFN-γ (10 µg/ml and 1 ng/ml respectively). (C) and (D) detection of protein levels of MIP-1α and TNF-α, respectively in supernatants of J774A.1 cell cultures. Gene expression and protein levels were determined at the late time point (72 h) after *L. donovani* infection. The relative expression of transcripts are expressed as the mean ± S.D. of the three replicates from one biological experiment. Asterisks indicate statistically significant up-regulation in gene expression (p≤0.05) by infected J774A.1 cells stimulated with LieIF/IFN-γ as compared to cells treated with IFN-γ only. The protein levels in supernatants are expressed as the mean ± S.D of three separate experiments run independently in triplicates.

Chemokines and proinflammatory cytokines are involved in both innate and adaptive immunity, facilitating the initiation and maintenance of the immune response mechanisms that are elicited by intracellular parasites [38]–[41]. The chemokine MIP-1α has a key role in immunity against experimental visceral leishmaniasis [42]–[44]. Moreover, the proinflammatory cytokine TNF-α potentially plays a role as a triggering signal for NO generation [45]. The MIP-1α and TNF-α gene expressions in J774A.1 cells treated with LieIF/IFN-γ pre- or post-infection were determined by quantitative real-time PCR. The expression of GAPDH was used to normalise the data, and these results are presented in Figure 6. Infected cells pre-treated with LieIF/IFN-γ showed a clear 5-fold up-regulation in MIP-1α mRNA expression compared to infected macrophages treated only with IFN-γ (p<0.05) (Fig. 6A). A low level of MIP-1α expression was detected in macrophages that were incubated with medium only and cells pre-treated with LieIF alone exhibited low MIP-1α mRNA expression as well. In contrast, when J774A.1 cells were post-infection treated with LieIF/IFN-γ, no increased expression in MIP-1α was observed compared to infected macrophages treated only with IFN-γ. MIP-1α was also detected in culture supernatants at the late time point (72 h) after *L. donovani* infection. Pre-treatment of infected cells with LieIF/IFN-γ induced the release of MIP-1α. Nevertheless, control J774A.1 cells not exposed to LieIF/IFN-γ, as well as infected J774A.1 cells exposed to IFN-γ only, elicited a MIP-1α response of similar magnitude at 72 h after infection (Fig. 6C). In addition, infected J774A.1 cells that were pre-treated with LieIF/IFN-γ showed an 8.6-fold TNF-α up-regulation of gene expression, compared to infected cells treated only with IFN-γ (Fig. 6B). However, TNF-α release in supernatants of infected J774A.1 cells pre-treated with LieIF/IFN-γ was not significant when compared to control groups (Fig. 6D). Conversely, post-infection treatment of the cells with LieIF/IFN-γ did not induce any up-regulation in MIP-1α or TNF-α mRNA expression. Similarly, infected macrophages post-treated with LieIF/IFN-γ released similar levels of MIP-1α and TNF-α to the control groups.

### Correlation of TNF-α production with generation of ROS in parasitized J774A.1 cells

**Figure 7:**
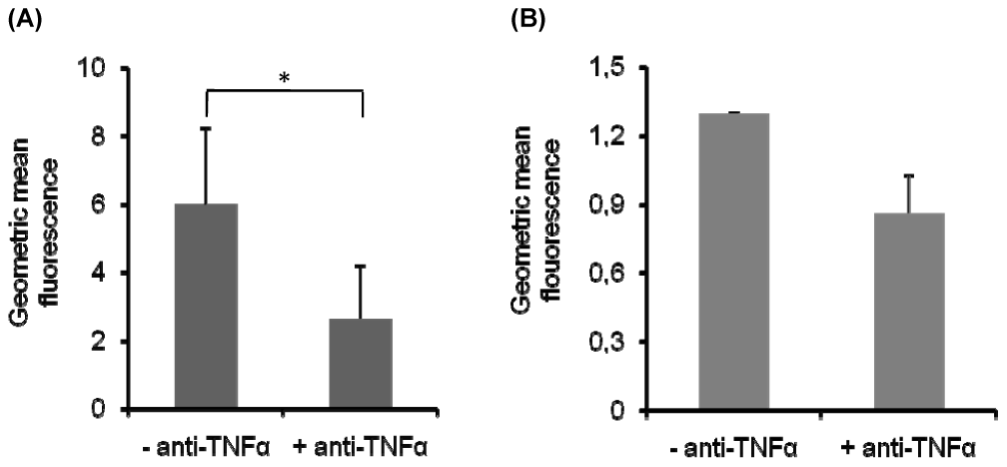
Correlation of TNF-α production with generation of ROS in parasitized J774A.1 cells. The effect of TNF-α on ROS production in J774A.1 cells pre- and post- infection treated (A and B, respectively) with recombinant LieIF/IFN-γ (10 µg/ml and 1 ng/ml respectively), with or without anti-mouse TNF-α monoclonal antibody (4 µg/ml), was determined at the late time point (72 h) after *L. donovani* infection. Cells were analyzed for intracellular ROS with FACS Calibur. All data are presented as the mean ± S.D. of three independent experiments. Data are expressed by the formula: Geo Mean  =  Geo Mean_(J774+LieIF+MON2+IFN-γ)_ – Geo Mean_(J774+MON2+IFN-γ)._

Nitrogen and reactive oxygen (ROS) intermediates are important in activation of transcription factor NF-κB, which controls the activity of numerous immunity and inflammation genes, including TNF-α [46]. In order to clarify the interplay between NO and ROS generation and TNF-α production, we tested the effect of anti-TNF-α monoclonal antibody on oxidative burst in parasitized macrophages, at the late time point (72 h) after *L. donovani* infection. J774A.1 cells, pre- infection treated with LieIF/IFN-γ combined with anti-TNF-α monoclonal antibody produced significantly lower amounts of ROS compared to J774A.1 cells pre- infection treated with LieIF/IFN-γ only (p = 0.003) (Fig. 7A). Nevertheless, TNF-α neutralization had no effect on ROS production in J774A.1 cells that were post- infection treated with LieIF/IFN-γ (p = 0.121) (Fig. 7B).

## Discussion

Macrophages are phagocytic cells that play a critical role in host immune responses to microbial infection. They play a pivotal role in initiating protective immune response to *Leishmania* infection whereas they allow the growth of this intracellular pathogen. During the past decades, a large body of evidence has supported the notion that the IFN-γ cytokine plays a decisive role in anti-leishmanial defence [47], [48]. Upon IFN-γ activation, macrophages provide the necessary regulatory signals in the form of cytokines to induce T cell activation and elicit several effector mechanisms involved in the control of infection. In IFN-γ- primed bone marrow-derived macrophages, *L. donovani* promastigotes induce the secretion of NO, which plays an essential role in the control of *Leishmania* infection by these macrophages [49], [50]. However, despite the importance of IFN-γ in the development of resistance to *Leishmania* infection, it was reported that the administration of exogenous IFN-γ does not protect susceptible BALB/c mice from *L. major* infection [51]. In addition, IFN-γ mRNA was detected in the infected skin lesions of not only resistant C57BL/6 mice but also susceptible BALB/c mice [52]. These reports suggest that IFN-γ alone is not sufficient to control *L. major* and that additional factor(s) would be required for the development of protective immunity to *Leishmania* infection. Exosomes of *Leishmania* are considered to play a role during the early stage of infection by delivering pre-emptive strikes that create permissive environment for infection with particularly a role for gp63, the major promastigote surface glycoprotein, and EF-1α, elongation factor 1α, which are delivered into the cytosol of the infected macrophage where they activate multiple host protein-tyrosine phosphatases. This way, these proteins negatively regulate IFN-γ signalling pathways and thus prevent the effective expression of microbicidal functions of the macrophage [53]. LeIF, antigen well known for its immune-modulating activities and ability to induce secretion of IL-12 cytokine from macrophages or monocytes of healthy donors [18], [19] or from PBMC from either leishmaniasis patients or normal individuals [16], constitutes an abundant protein in the exosomes of *L. donovani* stationary phase promastigotes [18] that is supposedly also delivered to infected cells.

In this study, we demonstrated the synergistic effect of the recombinant proteins LieIF and IFN-γ in eliminating intracellular *L. donovani* parasites in an *in vitro* macrophage infection model thus inferring a potential role for this exosomal protein during the early stages of infection. Our findings suggest that treatment of J774A.1 macrophages with LieIF/IFN-γ, prior to *Leishmania* infection, led to a statistically significant reduction in intracellular parasite growth at the late time point of infection (72 h). This result was confirmed by the significant reduction in both infection rate and parasite load. The observed immunomodulatory activities of IFN-γ are consistent with previous studies, and our results demonstrate clearly that LieIF reinforces the activities of IFN-γ [54]. The finding that LieIF/IFN-γ- pre-treated J774A.1 cells showed resistance to *L. donovani* infection prompted us to examine the efficacy of LieIF on intracellular growth of *L. donovani* following infection, and we showed that J774A.1 cells treatment with LieIF/IFN-γ after *Leishmania* infection also led to a significant prevention of parasite growth at early (19 h) and late time (72 h) points of infection.

Here, reduction of intracellular parasite growth upon LieIF/IFN-γ treatment was shown to be mediated by the production of anti-microbial effector molecules. Nitric oxide and oxidative mediators are proven leishmanicidal molecules capable of killing parasites effectively upon exposure to Th1 cytokines, including IFN-γ and TNF-α. The role of NO in controlling *Leishmania* MΦ infection is well established in humans and other mammals [48], [55]–[61]. In particular, NO release has been documented in human PBMC-derived MΦs infected with *L. infantum in vitro.* This production was increased after activation with IFN-γ and bacterial LPS and was correlated with leishmanicidal ability [62]. In our study, treatment of J774A.1 cells with LieIF/IFN-γ prior to *L. donovani* infection led to significant production of NO as compared to J774A.1 cells treated with IFN-γ alone, at both early (4 h) and late time points (72 h) after infection, suggesting this pre-infection treatment induced resistance to *L. donovani* infection in an NO-dependent manner. Interestingly, J774A.1 cells treated with LieIF/IFN-γ after their infection produced greater levels of NO both at early (19 h) and late time points (72 h) of infection. Collectively, these results suggest that treatment with LieIF/IFN-γ, either pre- or post-infection, impairs *L. donovani* macrophage infection in an NO-dependent manner.

In addition to NO production, activation of macrophages leads to ROS production through an oxidative burst that is ultimately responsible for the leishmanicidal activity [61], [63]–[65]. Although ROS derivatives normally lead to the destruction of the phagocytosed microorganism, *Leishmania spp*. have been adapted to survive and replicate in this hostile environment by deploying antioxidant systems or by suppressing macrophage ROS production [63]. *Leishmania* inhibits the production of O_2_^−^ *in vivo*, and it has been reported that O_2_^−^ and H_2_O_2_ levels are significantly lower in monocytes from patients with active visceral leishmaniasis as compared to healthy controls [66], [67]. To assess whether the intracellular parasite growth inhibition is also mediated by ROS derivatives, we determined ROS production at the late time point (72 h) after infection, when the inhibition of intracellular parasite growth was most prominent. Our results support the notion that ROS production is responsible for leishmanicidal activity. Indeed, we observed that J774A.1 cells treated with LieIF/IFN-γ, pre- or post-infection, led to a significant increase of ROS generation which suggests that LieIF, in synergy with IFN–γ, is able to confer protection to *L. donovani* infection by driving the innate immune response towards the up-regulation of NO and ROS, the potent macrophage-derived microbicidal molecules that are critical in controlling *Leishmania* infection [68], [69].

Chemokines are structurally defined small proteins that are the key molecules in recruiting immune cells by chemotaxis; they also act in leukocyte activation, inflammatory diseases and anti-microbial mechanisms [39], [70]. Furthermore, they have a much wider range of biological activity, such as participation in cell-mediated immunity and involvement in Th1-Th2 differentiation, which is of the greatest importance in the control of infection by intracellular protozoa [71]–[73]. The CC chemokines (MIP-1α, MCP-1) play an important role in restricting disease progression in leishmaniasis [12]. In this study, we focused on MIP-1α because it has been shown to have an important role in limiting macrophage parasitic burden [11]. MIP-1α treatment in *L. donovani*-infected macrophages induces the production of the protective Th1-type cytokine IL-12 and inhibits the production of the non-protective Th2-type cytokines, IL-10 and TGF-β [12]. Our study confirms previous findings that MIP-1α treatment restricts the parasite burden via the induction of the superoxide anion and nitrite generation in *L. donovani*-infected macrophages [74]. Thus, our findings clearly showed that *L. donovani*-infected J774A.1 cells pre-treated with LieIF/IFN-γ exhibited a 5-fold up-regulation in MIP-1α mRNA expression at the late time point (72 h) of infection. It is noteworthy that at the same time point, these cells presented significant intracellular parasite growth inhibition in a NO- and ROS- dependent manner. The levels of MIP-1α were also detected in culture supernatants of J774A.1 cells. Despite the observed up-regulation of MIP-1α mRNA expression in J774A.1 cells pre-treated with LieIF/IFN-γ, we did not detect significant different protein levels among the tested groups. This is in accordance with reports regarding poor correlation between the level of mRNA and level of protein [75].

MIP-1α is the protein primarily responsible for stimulating TNF-α secretion by macrophages [76]. TNF-α provides a second signal to induce microbicidal activity in IFN-γ-activated macrophages by stimulating NO production [48]. Our results showed that *L. donovani*-infected J774A.1 cells pre-infection treated with LieIF/IFN-γ had a 9-fold up-regulation in TNF-α mRNA expression at the late time point (72 h) of infection. It is worth noting that J774A.1 cells post-infection treated with LieIF/IFN-γ did not have elevated expression levels of MIP-1α and TNF-α mRNA. Similarly, no significant levels of TNF-α were detected.

In summary, we have demonstrated that J774A.1 macrophages treated with LieIF/IFN-γ prior or post their infection, inhibit the intracellular growth of *L. donovani*. In the case of pre-infection treatment with LieIF/IFN-γ and at 72 h post infection, J774A.1 cells eliminate the intracellular parasites by engaging a NO- and ROS-mediated leishmanicidal mechanism. We also showed, in accordance with other reports [10], that there is interplay between oxidative burst (ROS production) and TNF-α. Conversely, in the case of post-infection treatment with LieIF/IFN-γ, J774A.1 cells also inhibit the intracellular parasite growth in a NO- and ROS-dependent manner, but we found no correlation with the production of MIP-1α and TNF-α. These results indicate that post-infection treated J774A.1 cells engage NO- and ROS-mediated leishmanicidal activity probably by employing a mechanism independent of MIP-1α and TNF-α. Overall, the effective intra-macrophage parasite growth inhibition and the apparent immune-modulatory effect of LieIF pave the way for its evaluation as a candidate anti-leishmanial molecule.
